# A heel-strike real-time auditory feedback device to promote motor learning in children who have cerebral palsy: a pilot study to test device accuracy and feasibility to use a music and dance-based learning paradigm

**DOI:** 10.1186/s40814-018-0229-0

**Published:** 2018-01-29

**Authors:** Jaswandi Tushar Pitale, John H. Bolte

**Affiliations:** 1 The Ohio State University, Columbus, OH 43210 USA; 2 Bertec Corporation, Columbus, OH 43229 USA

**Keywords:** Auditory feedback, Pediatric cerebral palsy, Music and dance, Motor learning, Promoting heel strike, Toe walking, Rehabilitation

## Abstract

**Background:**

Cerebral palsy (CP) is a developmental disorder of movement and posture that occurs due to damage to the developing nervous system. As part of therapy, wearable sensors that trigger interactive feedback may provide multi-sensory guidance and motivation. A prototype of a heel-strike real-time feedback system has been developed which records the number of heel strikes during gait and indicates successful heel contact through real-time auditory feedback. The first aim of this feasibility study was to test the prototype accuracy.

Since the end user for this device is a child, the device should be esthetically appealing and sufficiently motivating for children to perform repetitive challenging therapeutic movements. The second aim of this study was to collect feedback from the subjects with regard to the device usability and understand if the bell sound used as feedback used was motivating enough for children to continue using the prototype. This would help us in developing the next generation of the device.

**Methods:**

The prototype was tested with typically developing children and children who have CP. The accuracy in detecting heel strikes was calculated. As part of the study, the subjects were also asked questions to test the device compliance and acceptability of the musical beats with the pediatric population.

**Results:**

The device accuracy in identifying heel strikes is 97.44% (95% CI 96.31, 98.88%). The subjects did not show any hesitation to put on the device and the sound feedback motivated them to move. Based on this pilot study, a minimum age limit of 5 years is appropriate and the intervention study should be conducted for no more than 30 min per week.

**Conclusions:**

The pilot study showed that a main study can be conducted to test auditory feedback as an intervention to promote motor learning in children who have cerebral palsy. No adverse event or safety issues were reported in the feasibility study.

**Electronic supplementary material:**

The online version of this article (10.1186/s40814-018-0229-0) contains supplementary material, which is available to authorized users.

## Background

Cerebral palsy (CP) can be described as a disorder of posture and movement, such as gait, that is caused by damage to the developing nervous system, before or during birth or in early months of infancy [1]. CP is among the most common pediatric neurological disorders in the US [2], affecting between 1.5 and more than 4 per 1000 live births [3]. In 2008, 30.6% of the children having CP had limited or no walking ability [3]. In the US alone, the cost of care for individuals with CP is estimated at about $8.2 billion of which a considerable amount can be attributed to gait deficits [2].

The rehabilitation treatments used by therapists to treat cerebral palsy are based on concepts of motor learning, such as active participation of the person with the disability, restoring functionality of the paretic limb, and repetition of voluntary movements [4]. Toe walking, foot drop [5], and walking manipulation [6] are some of the walking disorders that can arise due to cerebral palsy. Treatments are based on gait assessments in clinical labs, followed by physical therapy.

Children with CP, who have similar gross motor capability, perform differently in different environment settings [7]. Under clinical settings, due to the equipment, therapists, and clinicians, children may alter their gait and not show natural gait. This can make it difficult to accurately represent biofeedback during therapy. In such cases, personally engaging rehabilitation technology in facilitating home therapy could help increase the patient involvement in the therapy sessions as well as generate data to enable documentation of progress across weeks or months of home exercise performance. Using musical cues related to a dance-based learning paradigm might help address engagement of children with gait pathologies related to CP and other disorders.

**Fig. 1:**
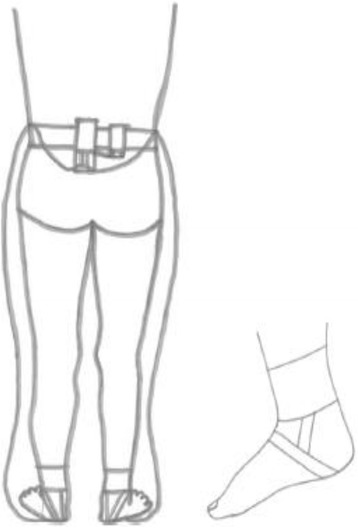
Sketch of subject wearing prototype of heel-strike real-time feedback device (l), heel cap, and sleeve attachment (r)

While wearable sensors delivering auditory feedback have been used to treat toe-walking in children and were found to improve heel contacts by 42% [8], previous designs were attached to or mounted in shoes/footwear [9–15] thus precluding their use during barefooted therapeutic training. Barefoot walking is a therapeutic technique used to address sensory acuity for children with CP [16]; thus, there is a need for a biofeedback system that participants may use to practice therapeutic activity while barefoot. Towards this end, a prototype of a heel-strike real-time feedback system was developed (Fig. 1). It consists of a force-sensing resistor, FSR 402 short from Interlink Electronics (California, USA), attached to a heel cap which was strapped on to the subject’s heel. To experience barefooted therapy sessions, the material used on which the sensor was attached to and which was under the foot of the child, was minimum, yet durable enough to protect the sensor and not damage it. The device was powered using a 9-V battery. When the heel touched the ground, it was detected by device in real-time and a bell sound was played. Some forms of dance such as Indian dance use bells around the ankles to keep the artist informed about the beat and rhythm. Music and dance as an intervention in rehabilitation sciences have been associated with neuroplasticity and that have been the inspiration behind this prototype development.

A randomized controlled trial is needed to test the efficacy of using this prototype along with auditory feedback to promote heel strikes in children with CP. But, one of the difficulties faced while testing with this age group is that gait training can be frustrating for them and that they lack motivation [17]. This might be the reason that compared to adults, there are lesser studies showing feasibility of wearable sensors used for movement retraining for children with walking disorders [14, 18, 19]. Among them, there has been only one instance of testing the device at home [14]. A feasibility study was thus necessary to be conducted with the target audience that is children, before a full-scaled RCT can be conducted to test the efficacy of using the developed prototype and auditory feedback.

To evaluate the efficacy of using the prototype in promoting heel strikes through biofeedback, the device must record them and be accurate in measuring the number of heel strikes. The first aim of this feasibility study was to test the prototype accuracy.

Since the end user for this device is a child, the device should be esthetically appealing and sufficiently motivating for children to perform repetitive challenging therapeutic movements. The second aim of this study was to collect feedback from the subjects about the device usability and understand if the bell sound used as feedback was motivating enough for children to continue using the prototype. This would help us in developing the next generation of the device.

## Methods

### Participants and recruitment

The device was tested with 11 children, of which 8 children were typically developing (TD) and 3 are with cerebral palsy (CP). The subjects were assented, and consent was obtained from at least one parent. The study was approved by the Office of Responsible Research Practices, OSU (IRB 2009B0396). Since this was a pilot study and the efficacy of an intervention was not tested, this was a convenience sample. Both, typically developing and with CP children were tested to observe if there was any distinguishable effect of the device between the two groups.

The subjects were asked to remove their footwear and given the choice of keeping their socks on. The heel-strike real-time feedback system was put on their feet.

In case of children having CP, normal heel-toe gait is a movement pattern that they are not accustomed to. This can be analogous to TD children performing some novel movement patterns. The TD children were taught four movements and allowed to practice them before putting on the heel-strike real-time feedback system. For all the steps, the initial position was two feet together with feet flat on the ground. Except for the first step, all the other steps were performed in counts of 4. For all the steps, the subjects were also asked to pick up their foot and place it at the required place without dragging the foot. These movement patterns were inspired by Indian dance movements. The description of the four movement patterns is as below:

- Step 1—Foot tap in the place by alternating feet. The feet were to be placed flat on the ground.
- Step 2—At count 1, either left or right foot was placed in the front with the ankle dorsiflexed and the heel touching the ground. At count 2, the dorsiflexed foot was brought back to the initial position, i.e., with both the feet together. While the foot was being placed in the front and brought back together, weight distribution of the body changed from being supported by two feet to one foot until the heel of the other foot touched the ground. At count 3, the other foot which was stationary was placed in the front and then brought back to initial position at count 4.
- Step 3—The third step was like the second, the only difference being that at count 1, instead of placing the heel of one foot in the front, it was placed at the side. At count 2, the subject brought the foot back to the initial position. Count 3 was the other foot at the side.
- Step 4—At count 1, the subject was asked to pick up one of his/her foot and place the toe behind the heel of the other stationary foot and at count 2 bring the foot back to the initial position. At count 3, the toe of the other foot went behind the heel of the first foot. The instructions given to the subject were that at count 1 and count 3, the toe should be touching the ground, not the heel of the foot that was moving.

The details of the TD subjects (H1, H2, H3, H4, H5, H8, H9, H10), subjects having CP (E1, E2, E3), and the movements performed by each are given in Table 1. A threshold of 50% of the maximum sensor value was set to detect heel strikes. While the subjects performed these movements, videos were recorded and the software recorded the number of heel strikes on each foot.

**Table 1:** Subject information

Subject	Age (years)	Walking disorders	Number of trials analyzed	Movements performed
H1	8	TD	8	Indian dance steps
H2	9	TD	7	Indian dance steps
H3	6	TD	8	Indian dance steps
H4	8	TD	6	Indian dance steps
H5	9	TD	8	Indian dance steps
H8	9	TD	6	Indian dance steps
H9	9	TD	7	Indian dance steps
H10	7	TD	8	Indian dance steps
E1	4	Walks pronated	3	Walking independently, Side stepping
E2	12	Needs an assistive device to walk	2	Walking with support
E3	13	Walks independently, occasionally walks on her toes	3	Walking independently

### Primary outcomes

The recorded videos were observed. If a sound was produced when the subject touches the heel to the ground, it was counted as a true positive (TP). If no sound was produced when the toe touched the ground, it was counted as a true negative (TN). If any double sounds were produced when the heel touched the ground or if there were sounds when the toe touched the ground, it was counted towards false positives (FP). If no sound was produced during heel strike, it was counted towards false negatives (FN). Performance of the heel-strike real-time feedback system was also assessed on calculating the sensitivity and the specificity of the system for each subject.

The system accuracy was calculated using Eq. (1).

1 $$ A\mathrm{ccuracy}=\frac{\left(\mathrm{true}\  \mathrm{positives},+,\mathrm{true}\  \mathrm{negatives}\right)}{\begin{array}{c}\Big(\mathrm{true}\  \mathrm{positives}+\mathrm{true}\  \mathrm{negatives}+\\ {}\mathrm{false}\  \mathrm{positives}+\mathrm{false}\  \mathrm{negatives}\Big)\end{array}} $$

2 $$ \mathrm{Sensitivity}\  \mathrm{for}\  \mathrm{subject}\kern0.5em =\frac{\mathrm{sum}\ \mathrm{of}\  \mathrm{true}\  \mathrm{positives}}{\mathrm{sum}\ \mathrm{of}\  \mathrm{true}\  \mathrm{positives}+\mathrm{sum}\ \mathrm{of}\  \mathrm{false}\  \mathrm{negatives}} $$

3 $$ \mathrm{Specifity}=\frac{\mathrm{sum}\ \mathrm{of}\  \mathrm{true}\  \mathrm{negatives}}{\mathrm{sum}\ \mathrm{of}\  \mathrm{true}\  \mathrm{negatives}+\mathrm{sum}\ \mathrm{of}\  \mathrm{false}\  \mathrm{positives}} $$

Only descriptive statistics are available for these performance measures.

### Secondary outcomes

The second aim of this pilot study was to observe the trials and the subject for device compliance and assess acceptability of the musical beats. Subjects were encouraged to move around the room with the heal-strike real-time sensing device and asked open-ended questions such as “How do you feel?,” “Do you like the sound?,” and “Would you like to wear this for a prolonged period?” Other parameters observed were fatigue, boredom, and safety of using the device. This pilot study will also help us design the inclusion and exclusion criteria for recruitment for the main study.

## Results

In total, 66 trials and 1002 heel strikes were analyzed across 11 subjects. Table 2 gives the average accuracy for each subject.

**Table 2:** Device accuracy

Subject	Total number of heel strikes	Percentage of missed heel strikes	Percentage of false heel strikes	Average accuracy (percentage)
H1	111	2.70	1.80	95.97
H2	107	0.94	0	99.56
H3	77	1.30	10.39	90.11
H4	65	0	1.54	99.3
H5	101	0	0.99	99.14
H8	89	0	4.50	97.81
H9	104	0.96	0	99.18
H10	155	0	0	100
E1	144	2.78	4.86	92.72
E2	28	0	3.57	96.55
E3	21	4.76	0	94.84

### Primary outcomes

**Fig. 2:**
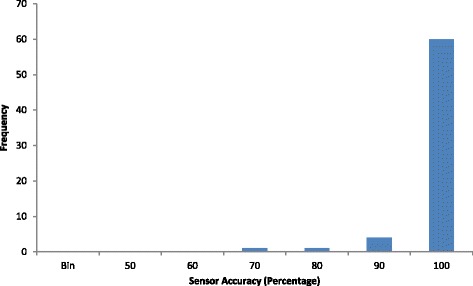
Histogram of accuracy of detecting heel strikes

The histogram of the accuracy in Fig. 2 shows that the accuracy is skewed left with mode at 100% with the mean being 97.44% (95% CI 96.31, 98.88%). Most of the observations were greater than 80% except one outlier at 70%.

**Fig. 3:**
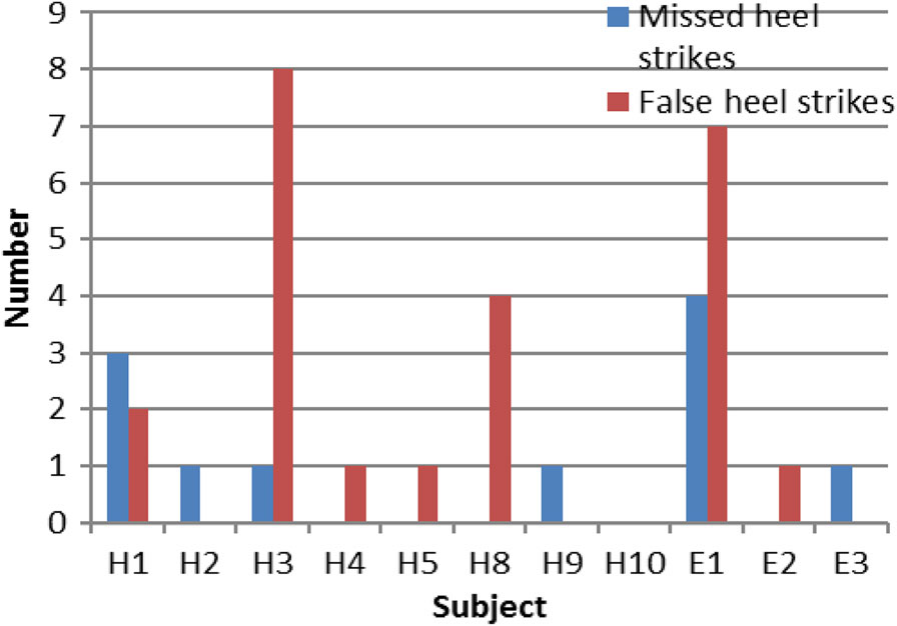
Bar graph showing false heel strikes and missed heel strikes

The bar graph in Fig. 3 shows the sum of the total missed heel strikes and false heel strikes detected by the sensor for each subject.

**Fig. 4:**
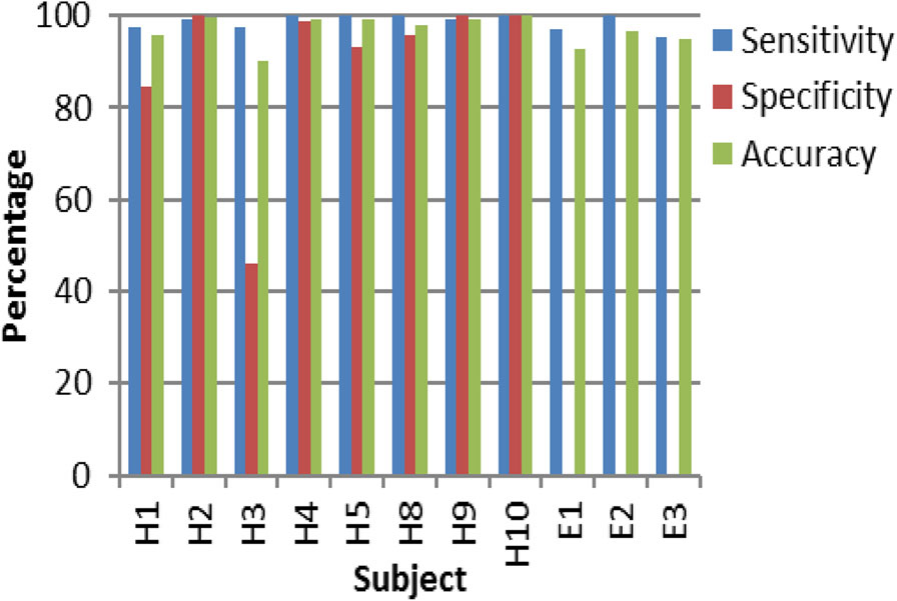
Sensitivity, specificity, and accuracy of the device

Figure 4 shows the accuracy (Eq. 1), sensitivity (Eq. 2), and specificity (Eq. 3) of the heel strike sensing system for each subject.

### Secondary outcomes

#### Device compliance and acceptability of musical beats

The subjects liked the sound of the bells and did not hesitate to put the sensors on. In most of them, the sound encouraged them to move around in a way that would trigger the sensors and cause them to make sound. Most often, after the trials, they would request for one of their favorite songs and try to move around the room with some free style dance movement. Some children would even try to maintain the rhythm of the bell sound with the music being played. In other words, the sensors encouraged them to impact their heels with a specific rhythmic movement. All the children responded positively to the open-ended questions, only one subject showed some initial inhibitions towards the wires going from the sensor attached to the heel to the digitizer board attached to the waist.

The time to put on the system was not a barrier, and it provided means of therapy and physical exercise at home. This is an attractive option to practice skills necessary for motor learning [20]. If therapy is conducted at home, parents could get involved during the rehabilitation process which can be a benefit [21]. The system was tested both on carpet and tile and did not show any difference in the accuracy based on the surface.

#### Safety

The subjects did not experience any adverse effects or safety issues after wearing the heel-strike real-time feedback system. They were encouraged to move around as much with the prototype device, and there were no issues such as slipping, tripping, or falls observed. No subject expressed any concern over the safety of the prototype or withdrew from the study due to concerns about the safety.

#### Recruitment and retention

Parent/s of 11 healthy children and 4 children with CP responded positively to the recruitment email and flyer. Among the 11 healthy children, 3 of them did not complete the trials. Two of them were not interested in complying with the test protocol and one of them was too young (4 years) to take orders. There was no noticeable fatigue seen among the children after the intervention. The movements used were easy to imitate.

Before beginning the trials, the parents were asked if their child had any prior experience with music, dance, or any other form of activity which could indicate that they had been introduced to the concept of rhythm. Among the 11 participants, only 2 mentioned having such experience. Among these 2, one child completed the protocol while the other was not interested. For the child who left the trial, it could signify that there was lack of interest because the child was too familiar with a similar protocol or sound. This could indicate that one type of sound can get monotonous and future generations of the device should have capability to give the user the option to choose a sound of his/her choice or implement higher complexity of sound feedback by altering the pitch or volume based on the movement performance. For the child with a dance training who completed the protocol, it was observed during free style movement that the child had a highly elevated level of rhythmic sense. Previous training in dance or music can bias the results of the future study.

## Discussion

The pilot study evaluated the feasibility of the prototype and the methods used for conducting a RCT. The lowest accuracy was shown by subject H3 (Fig. 4). Figure 3 shows that the poor performance of the sensor in case of low accuracy can be attributed more towards false heel strikes than missed heel strike detection. Figure 4 shows that in nine out of 11 subjects, the sensitivity of the system is either higher than or equal to the specificity of the system. This indicates that the device was better in identifying positive heel strikes than neglecting the false heel strikes. This is a limitation of force-sensing resistors [22, 23], making the device over-sensitive at times. The sensitivity of the device could be reduced by decreasing the sampling rate or by introducing a pause while acquiring sensor signals. If the pause is large, this would impose additional constraints on the subject which has been criticized in the past [24].

The data shows that the accuracy is 100% for the last trial for each healthy subject. This could indicate that over time, the subjects learned to place their foot in such a way as to avoid the double sounds caused due to weight change or rolling of the heel. The device thus could potentially be used as a learning system in gait retraining. One of the applications of the heel-strike real-time feedback system is to use it as an application of interactive arts and provide knowledge of performance and knowledge of results. This may help in solving the issue of patient engagement in pediatric population and simultaneously document the amount of activity performed [24]. With the help of this device, the children could focus on therapy with better attention and perform repetitive movements which are important to show improvements in motor skills [20]. The prototype can be used to test the hypothesis that, auditory feedback can be used in gait retraining for children who have CP and abnormal gait patterns.

In our testing protocol, each trial represented a different movement pattern. Also, among the children with CP who were recruited for the test, gait pathologies and its degree varied; two of the children walked independently while one of them required an assistive device to walk with. This benefitted the initial trials by helping the researchers understand requirements of a broader target group for developing the product prototype. However, the accuracy showed no trend across trials or subjects which indicates that the accuracy of the heel-strike real-time feedback system was independent of the subjects, the foot on which sensor was placed, and the gait pathology. The system thus can be used on a wide range of children irrespective of their walking patterns, not limiting the recruitment to gait pathology. While designing RCTs to test the efficacy of the therapy intervention, the subjects should be categorized as per the GMFCS level [25] to help understand the effect of the intervention and its future prescription.

Based on the age distribution of the subjects for the pilot study and their response to the prototype and the trials, for the RCT, a minimum age limit of 5 years will be appropriate. Data collection will be difficult for children younger than 5. After observing the children during these feasibility trials, based on their attention span and interest, it is suggested that an intervention based session of 30 min, once a week will be feasible. To have an unbiased result of the RCT, an exclusion factor of dance or music training should be mentioned in the recruitment material.

One shortcoming of this feasibility test was that the device was tested only with three children having CP. The physical and mental conditions of children with CP can differ. Testing with more children with CP can help address a larger variety of anxieties and physical compliance issues that could arise. This can lead to optimizing the RCT design and further revisions of the device. Also, though barefooted therapy is the preferred choice, for some pathology, that may not be a possibility. Feasibility of using the device with orthotics or shoes was not tested. The high sensitivity of the sensor material may increase the number of false heel strikes in such cases and this needs to be tested and addressed.

## Conclusion

The pilot study showed that a main study can be conducted to test auditory feedback as an intervention to promote motor learning in children who have cerebral palsy. No adverse event or safety issues were reported in the feasibility study. The intervention would involve the bell sound that has been used in the pilot study and a combination of the basic steps described in the methods. The intervention would be tested on children above 5 years old and who do not have prior training in music or dance.

## Additional files

Additional file 1: Data for individual trials for typically developing children (XLSX 11 kb)

Additional file 2: Data for individual trials for children with CP (XLSX 10 kb)
